# Targeting purine metabolism in ovarian cancer

**DOI:** 10.1186/s13048-022-01022-z

**Published:** 2022-08-13

**Authors:** Jingchun Liu, Shasha Hong, Jiang Yang, Xiaoyi Zhang, Ying Wang, Haoyu Wang, Jiaxin Peng, Li Hong

**Affiliations:** Department of Obstetrics and Gynecology, Renmin Hospital of Wuhan University, Wuhan, China

**Keywords:** Purine metabolism, Ovarian cancer, Metabolizing enzyme, Antimetabolites, Purinergic signaling

## Abstract

Purine, an abundant substrate in organisms, is a critical raw material for cell proliferation and an important factor for immune regulation. The purine de novo pathway and salvage pathway are tightly regulated by multiple enzymes, and dysfunction in these enzymes leads to excessive cell proliferation and immune imbalance that result in tumor progression. Maintaining the homeostasis of purine pools is an effective way to control cell growth and tumor evolution, and exploiting purine metabolism to suppress tumors suggests interesting directions for future research. In this review, we describe the process of purine metabolism and summarize the role and potential therapeutic effects of the major purine-metabolizing enzymes in ovarian cancer, including CD39, CD73, adenosine deaminase, adenylate kinase, hypoxanthine guanine phosphoribosyltransferase, inosine monophosphate dehydrogenase, purine nucleoside phosphorylase, dihydrofolate reductase and 5,10-methylenetetrahydrofolate reductase. Purinergic signaling is also described. We then provide an overview of the application of purine antimetabolites, comprising 6-thioguanine, 6-mercaptopurine, methotrexate, fludarabine and clopidogrel. Finally, we discuss the current challenges and future opportunities for targeting purine metabolism in the treatment-relevant cellular mechanisms of ovarian cancer.

**Graphical Abstract:**

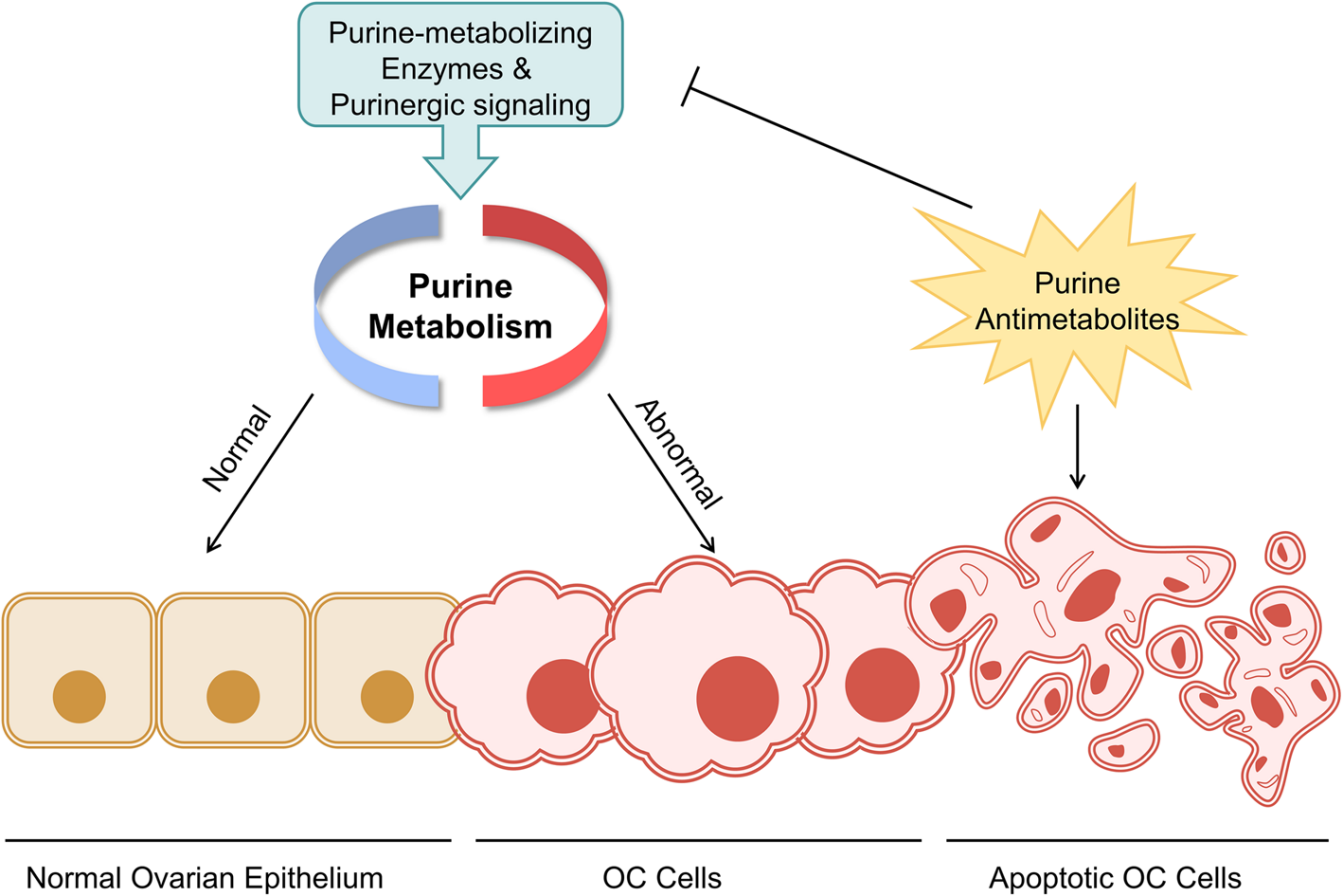

## Introduction

Ovarian cancer (OC) is the seventh most common cancer and the fifth leading cause of cancer-related death among women worldwide, with a 5-year relative survival rate of 49% [1, 2]. Primary debulking surgery and adjuvant platinum-based chemotherapy are the first-line standard-of-care treatments [3]. More than 70% of patients experience will relapse after first-line treatment and acquire drug resistance, which highlights the need for novel treatment options.

As one of the most abundant components in organisms, purine, in addition to forming DNA and RNA, is involved in the stabilization of immune regulation and the formation of energy carriers and functions as an essential cofactor in biochemical reactions, thereby influencing the growth of both cancer and non-cancer cells [4, 5]. The metabolic enzymes implicated in purine metabolism cause imbalances in purine pools that interfere with cell proliferation, migration and death [6, 7]. Furthermore, various purine antimetabolites exert antitumor effects through multiple mechanisms, such as direct toxicity, interference with the tumor microenvironment (TME), inhibition of DNA synthesis and interference with DNA damage repair [8–12]. Disorders of extracellular ATP (eATP), extracellular adenosine (eADO) and subsequent purinergic signaling also delineate pro-oncogenic or anti-oncogenic outlines [13]. Overall, purine metabolism is closely related to tumor progression.

This review presents recent reports of major purine-metabolizing enzymes in purine synthetic pathways (Table 1), outlines the multiplicity of purinergic signaling in OC development, gives an overview of the application of purine antimetabolites in OC, and discusses potential therapeutic strategies to target purine metabolism in OC.

**Table 1:** Summary of the main molecular features of purine-metabolizing enzymes associated with OC

Purine-metabolising Enzyme	Enzyme Number	Size/ Molecular Mass	Coding Gene	Gene Location	Subcellular Location	Function	Reaction in Purine Metabolism	Expression or Activity in OC	Ref
CD39	EC 3.6.1.5	510aa/58 kDa	ENTPD1	10q24.1	Plasma membrane, extracellular	Purine metabolism, Purinergic neurotransmitter regulation, Blocking platelet aggregation, Immunomodulation	eATP → eAMP	High	[14, 15]
CD73	EC 3.1.3.5	574aa/63 kDa	NT5E	6q14.3	Plasma membrane, extracellular, nucleus	Purine metabolism, Water-soluble vitamins and cofactor metabolism	eAMP → eAdo	High	[14–17]
ADA	EC 3.5.4.4	ADA1: 363aa/41 kDa	ADA	20q13.12	Plasma membrane, cytosol, lysosome, extracellular	Purine metabolism, Adenosine homeostasis, Immunomodulation	Adenosine → Inosine, Deoxyadenosine → Deoxyinosine	High	[18–20]
		ADA2: 511aa/59 kDa	ADA2	22q11.1	Extracellular, lysosome			High	
ADAR	EC 3.5.4.37	ADAR1:1226aa/136 kDa	ADAR	1q21.3	Cytoplasm, Nucleus	RNA editing	A-to-I RNA editing	High	[21–24]
		ADAR2:741aa/81 kDa	ADARB1	21q22.3	Cytoplasm, Nucleus			Unknown	
		ADAR3:739aa/81 kDa	ADARB2	10p15.3	Nucleus			Unknown	
AK	EC 2.7.4.3	AK4:223aa/25 kDa	AK4	1p31.3	Mitochondrion matrix	Purine nucleotide salvage, ATP level regulation	AMP + ATP ↔ ADP	High	[25–27]
		AK7:723aa/83 kDa	AK7	14q32.2	Cytoplasm, cytosol, Cell projection, cilium, flagellum		Adenosine → AMP	Low	
IMPDH	EC 1.1.1.205	IMPDH1:514aa/55 kDa	IMPDH1	7q32.1	Cytoplasm, Nucleus	Purine nucleotides de novo biosynthesis, Immunomodulation	IMP → XMP	Unknown	[28–31]
		IMPDH2:514aa.56 kDa	IMPDH2	3p21.31	Cytoplasm, Nucleus, cytosol			High	
PNP	EC 2.4.2.1	289aa/32 kDa	PNP	14q11.2	Cytoplasm	Pyrimidine metabolism, Purine salvage, Immunomodulation	Inosine → Hypoxanthine, Guanosine → Guanine, 2'-deoxyguanosine → Guanine, 2'-deoxyinosine → Hypoxanthine	Unknown	[32]
HPRT	EC 2.4.2.8	218aa/25 kDa	HPRT1	Xq26.2-q26.3	Cytoplasm	Purine salvage	Guanine → GMP, Hypoxanthine → IMP	Unknown	[33–35]
DHFR	EC 1.5.1.3	187aa/21 kDa	DHFR	5q14.1	Mitochondrion, Cytoplasm	Folate metabolism, Nitric oxide metabolism, Mitochondrial thymidylate de novo synthesis	Dihydrofolate → THF	High or Low	[36–39]
MTHFR	EC 1.5.1.20	656aa/75 kDa	MTHFR	1p36.22	Cytoplasm	Folate metabolism	Methylene-THF → Methyl-THF	Low	[40–43]

## Purine metabolism pathways

**Fig. 1:**
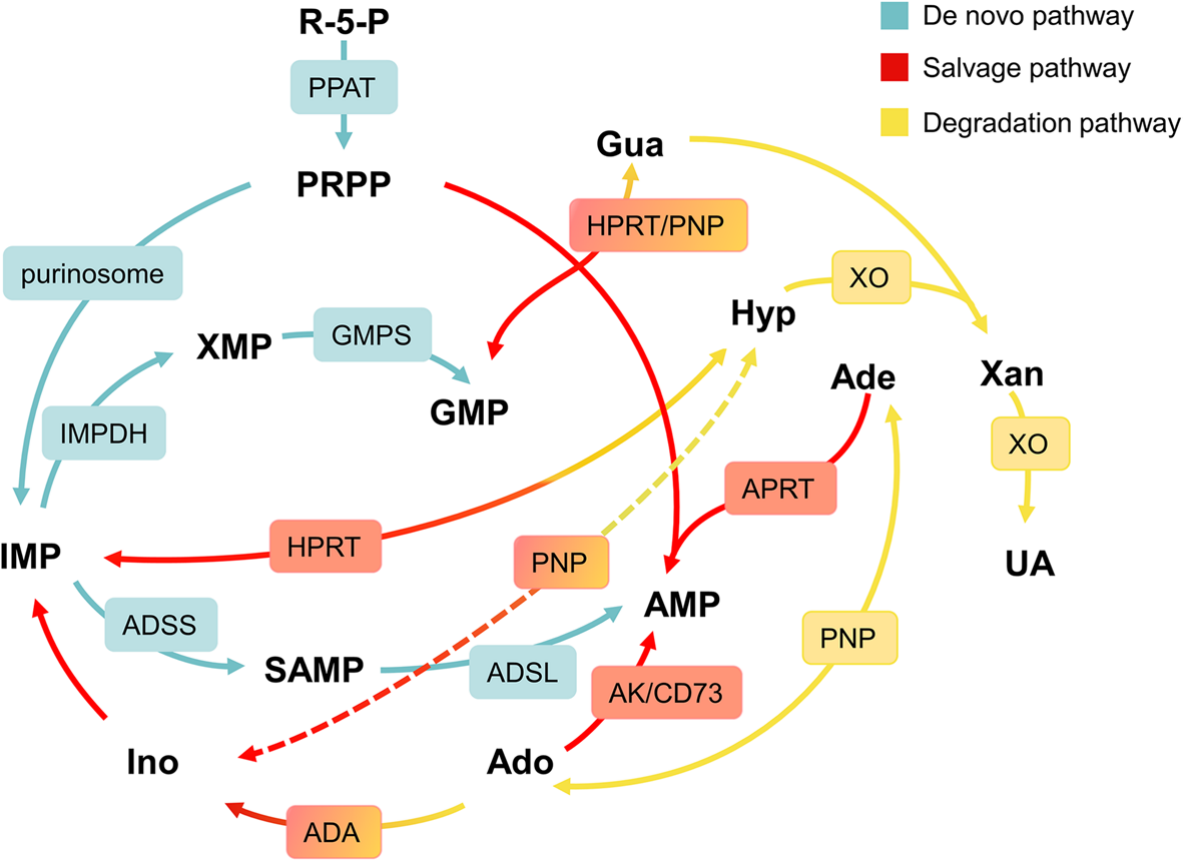
De novo, salvage and degradation pathways of purine nucleotides under the regulation of purine-metabolizing enzymes. The de novo pathway converts PRPP to IMP and, ultimately, GMP and AMP that further involve in nucleotide synthesis. The salvage pathway recovers purine bases and purine nucleosides to generate purine nucleotides. The degraded purine base becomes Xan with eventual conversion to UA. Cyan: de novo pathway; red: salvage pathway; yellow: degradation pathway; gradient color: involved in multiple metabolic pathways; arrows: purine metabolic pathways; squares: purine-metabolizing enzymes involved in related pathways. R-5-P: ribose 5-phosphate; PRPP: 5-phosphoribosyl-1-pyrophosphate; Gln: glutamine; THF: Tetrahydrofolate; Asp: aspartate; Hyp: hypoxanthine; Ino: Inosine; IMP: inosine monophosphate; Xan: xanthine; XMP: xanthosine monophosphate; Gua: guanine; GMP: guanosine monophosphate; Ade: adenine; Ado: adenosine; AMP: ado monophosphate; SAMP: succinyl-AMP; UA: uric acid; PPAT: phosphoribosyl pyrophosphate amidotransferase; IMPDH: IMP dehydrogenase; GMPS: GMP synthase; ADSS: adenylosuccinate synthase; ADSL: adenylosuccinate lyase; HPRT: Hyp Gua phosphoribosyltransferase; APRT: Ade phosphoribosyltransferase; ADA: Ado deaminase; AK: adenylate kinase; PNP: purine nucleoside phosphorylase; XO: xanthine oxidase

The stabilization of purine pools is determined by the balance between the synthesis and degradation of purine nucleotides (Fig. 1). The salvage pathway and de novo pathway are two different pathways for the synthesis of purine nucleotides in mammals. The salvage pathway recycles the degraded purine bases or nucleosides via 5-phosphoribosyl-1-pyrophosphate (PRPP) and catalysis by adenine phosphoribosyltransferase (APRT) and hypoxanthine guanine phosphoribosyltransferase (HPRT). There are other enzymes involved in the recovery of purines. Generally, this simple pathway meets most of the cellular requirements of a large percentage of normal cells with very low energy consumption [44, 45]. Rapidly dividing cancer cells rely more heavily on the de novo pathway, the basis for replenishing purine pools, to meet high energy demands [46]. The de novo pathway is triggered by a dynamic complex called purinosome [47]. Purinosome accumulates in the vicinity of mitochondria and microtubules to accelerate purine nucleotide synthesis by catalyzing the important step PRPP to inosine monophosphate (IMP) [48–50]. Two important rate-limiting enzymes, adenylosuccinate synthase (ADSS) and IMP dehydrogenase (IMPDH), catalyze the conversion of intracellular IMP to succinyl-AMP and xanthosine monophosphate (XMP) and then to AMP and GMP which are gradually degraded to xanthine and eventually hydroxylated to uric acid (UA) under the action of xanthine oxidase (XO) [51].

## Purine-metabolizing enzymes and mechanisms in OC

### Ectonucleoside triphosphate diphosphohydrolase and ectosolic-5′-nucleotidase

**Fig. 2:**
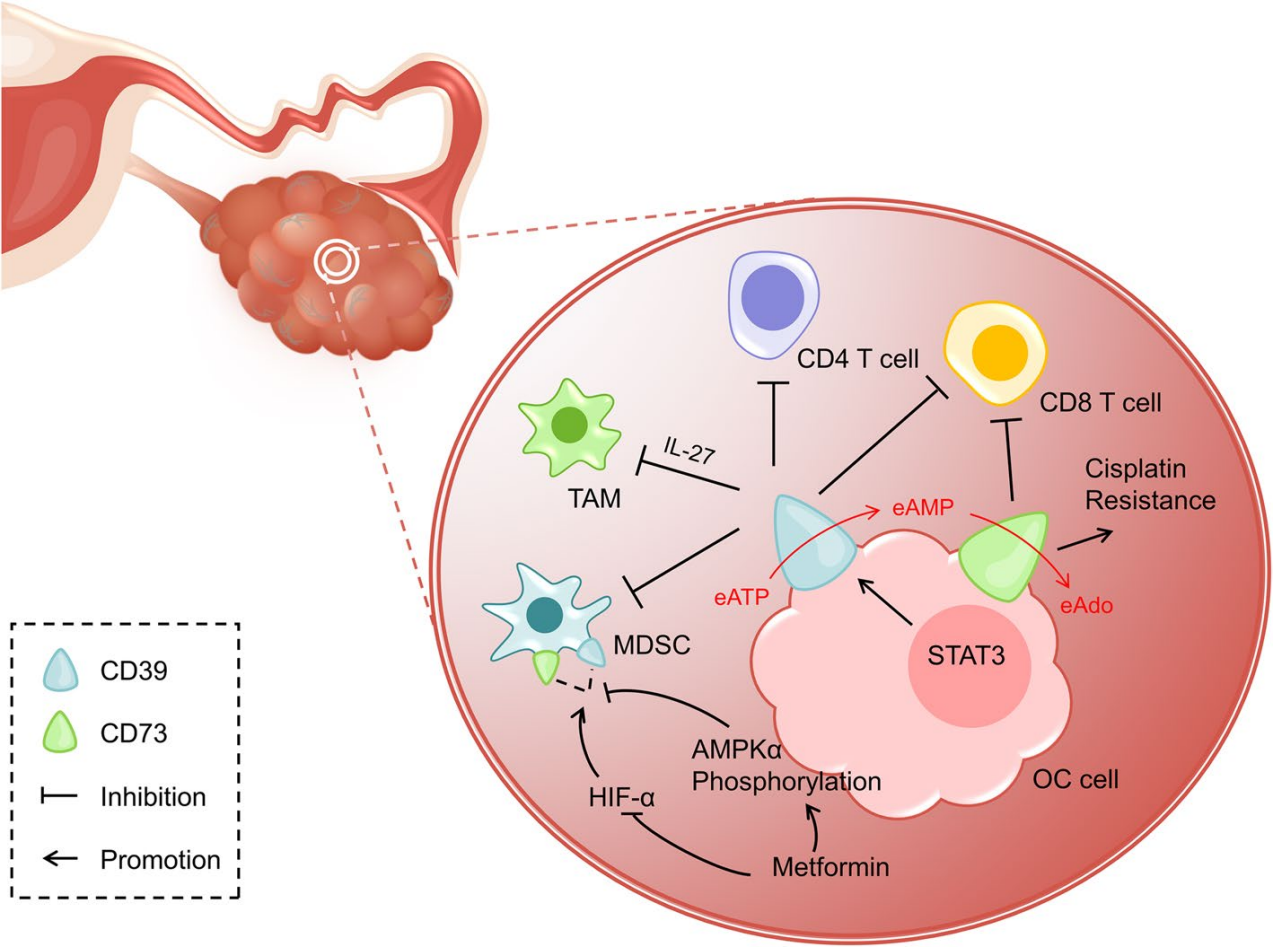
CD39 and CD73 in TME of OC. CD39 and CD73 localized on the surface of OC cells inhibit immune responses mediated by T cells, MDSC, and TAM in TME, and also induce cisplatin resistance. CD39 and CD73 dephosphorylate eATP to eAMP, ultimately converting it to eAdo. STAT3 induces cell surface acquiring CD39 in TME to promote immunosuppression. Metformin facilitates AMPKα phosphorylation and inhibits the HIF-α pathway to block the immunosuppression caused by high expression of CD39 and CD73 on MDSC. MDSC: myeloid-deriver suppressor cell; TAM: tumor-associated macrophage; TME: tumor microenvironment; eATP: extracellular ATP; eAMP: extracellular AMP; eAdo: extracellular Ado

Ectonucleoside triphosphate diphosphohydrolase (CD39, EC 3.6.1.5) and ectosolic-5′-nucleotidase (CD73, EC 3.1.3.5) are two membrane-bound ectonucleotidases. CD39 is the rate-limiting enzyme for the continuous dephosphorylation of eATP to extracellular AMP (eAMP) [52]. The 5'-nucleotidase activity of CD73 limits the rate of hydrolysis from eAMP to membrane-permeable eADO (Fig. 2) [53]. The eADO creates a highly immunosuppressive microenvironment by inhibiting the cytotoxicity of CD8 + T cells and NK cells while increasing activation of Treg cells and M2 macrophages [54–57]. This implies the balance between eATP and adenosine, which is jointly maintained by CD39 and CD73, is closely related to the immune-suppressive tumor microenvironment.

Traditionally, CD39 is considered as a contactor between immune cells. Tumor-reactive T cells can be identified by the expression of CD39 alone or the co-expression with CD103 [58, 59]. In recent years, it has been shown that CD39 is widely expressed in multiple human cancers, and high-level CD39 is found in M2-polarized tumor-associated macrophages (TAMs) in OC tissues [10, 14, 15, 60, 61]. STAT3 induces the acquisition of CD39 on cell surface in TME to suppress T cell response [62]. Noteworthy, it is IL-27 that mediates the immunosuppression with CD39 involvement, whose polymorphisms is related to the susceptibility to OC [10, 63]. Furthermore, CD73 is expressed on the surface of OC, and high-level CD73 appears to be significantly associated with poor prognosis in patients with high-grade serous OC, which, as mentioned previously, is probably because that CD73 indirectly suppresses CD8 + T cells and promotes immune escape [16].

Surprisingly, the study by Hoon Kyu Oh et al. found that patients with CD73 overexpression present more frequently with low stage, moderate differentiation, no lymph node metastasis, and negative cytologic results, which means that more CD73 may lead to a better prognosis [17]. A study regarding ovarian tumor-initiating cells found that CD73 is essential for OC initiation and growth, and is able to regulate tumor-initiating cells at the transcriptional level to promote expression of epithelial–mesenchymal transition (EMT)-related genes [64]. Besides, blocking CD73 reverses drug resistance in cisplatin-resistant OC cells [65]. Studies on other human tumors have reported that AKT signaling plays a role when CD73 promotes tumor progression and metastasis [66–69]. However, more mechanistic studies are needed in OC.

Current researches have noted that the anticancer effect of CD39 or CD73 inhibitors depends overwhelmingly on relieving T cell targeted immunosuppression and additionally on myeloid-deriver suppressor cells (MDSCs), which suggest CD39 and CD73 may serve as potential therapeutic targets for OC treatment [70–73]. A study found the antitumor activity can be mediated by blocking CD39 via eATP-P2X7-ASC-NALP3-inflammasome-IL18 pathway [74]. Besides, CD39 inhibitor POM-1 partially relieves the immunosuppressive function of TAMs [10]. As we would expect, anti-CD73 antibody reverses the immunosuppressive environment developed by docetaxel in OC but the inhibition of CD73 alone rather than CD39 is less effective [75]. Thus, the protocol for co-suppressing CD39 and CD73 is expected. An important finding is that metformin, the first-line drug for type-2 diabetes, blocks CD39 and CD73 by increasing AMPKα phosphorylation and inhibiting HIF-α pathway to interrupt immunosuppression caused by MDSC as well as enhance the anti-tumor activity of CD8 + T cells [14]. In addition, a pivotal immunosuppressive factor programmed death-1 receptor (PD-1) encourages tumor spread through interfering protective immunity [76]. Anti-PD-1 combined with anti-CD39 or anti-CD73 demonstrates a more pronounced slowing of tumor growth [74, 77]. In any case, there is still a long way to go to clarify the specific roles and mechanisms of CD39 and CD73, and deep studies will provide new insights into tumor immune networks.

### Adenosine deaminase

**Fig. 3:**
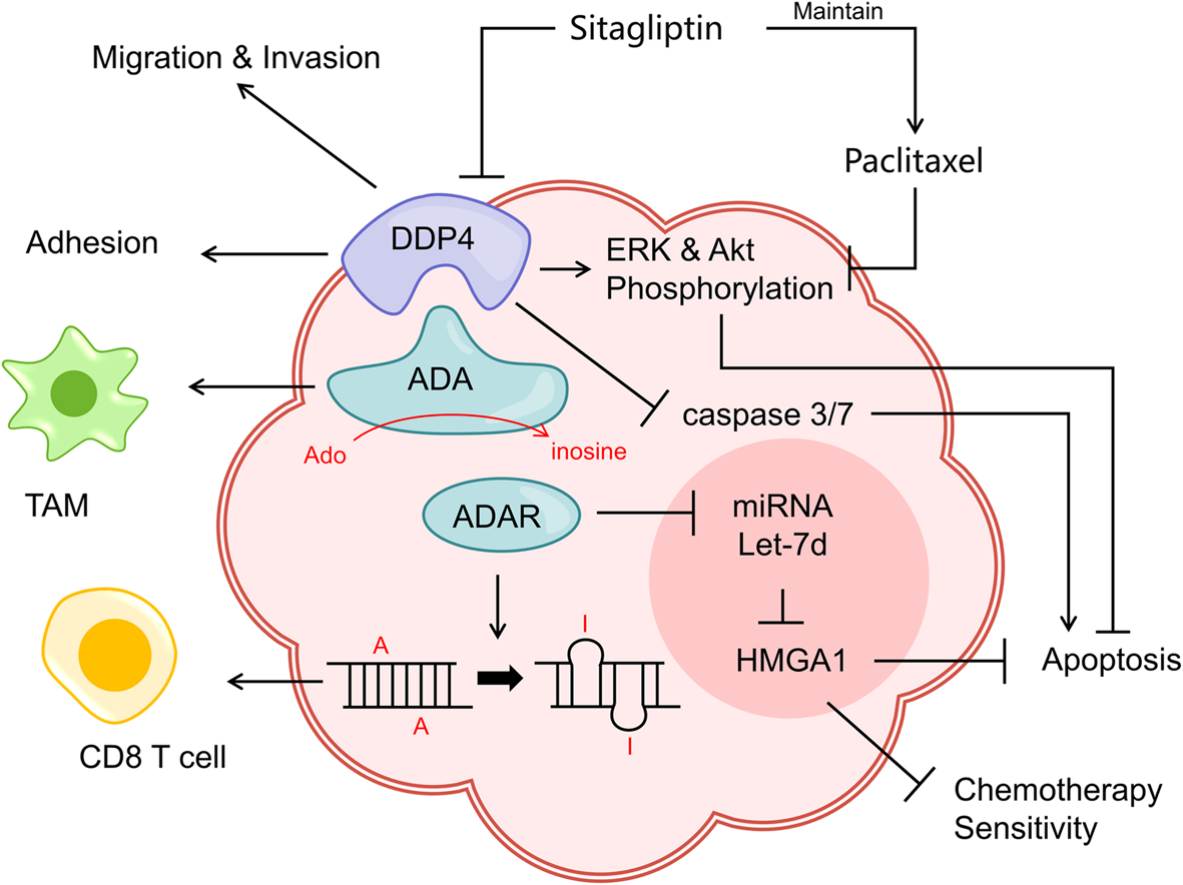
Role and mechanism of ADAR, ADA and its receptor DDP in OC. ADAR mediates A to I RNA editing to elicit CD8 T cell response and interferes with HMGA1 via miRNA Let-7d acting on OC apoptosis and chemotherapy sensitivity. ADA enhances the immune potency of TAM in TME with the capacity to convert Ado to inosine. DDP4, an important receptor for ADA, facilitates the migration, invasion and adhesion to mesothelial cells of OC. The DDP inhibitor Sitagliptin, increases caspase 3/7 activity to induce OC apoptosis on the one hand, and maintains the effect of paclitaxel on OC apoptosis via ERK and Akt pathways on the other hand

Adenosine deaminase (ADA, EC 3.5.4.4) catalyzes the irreversible hydrolytic deamination of adenosine and deoxyadenosine, the final products of which are inosine and deoxyinosine (Fig. 3) [18, 78, 79]. There are two isozymes of human ADA. ADA1 regulates adenosine concentration in the intracellular and interacts extracellularly with the adenosine receptor or dipeptidyl peptidase 4 (DDP4, EC 3.4.14.5) on the surface of immune cells [80, 81]. DDP4, expressed as a type II transmembrane protein, plays a pro-tumor role in a variety of human tumors and has the potential to act as a positive prognostic predictor [82]. It was reported that the interaction between ADA and DDP4 on the cell surface could regulate T cell activation and lymphocyte-epithelial cell adhesion [18, 83]. ADA2, with low abundance in humans, is mainly found in serum and its deficiency is associated with autoinflammatory diseases [84–87].

ADA level is significantly elevated in the serum and peritoneal fluid of OC patients and is positively correlated with pathological subtype and grade [19]. This may be a self-rescue measure in response to apoptosis caused by accumulation of toxic substrates adenosine and deoxyadenosine. D'Almeida SM et al. found that ADA, similar to CD39 inhibitors, partially blocked immunosuppression of TAMs [10]. At the same time, as a receptor of ADA, DDP4 is overexpressed strongly and the expression level is associated with FIGO stage or lymph node metastasis [88]. Besides, DDP4 increases the adhesion potency of OC to mesothelial cells with the participation of fibrin, and blocking DDP4 significantly inhibits cancer migration and invasiveness [89]. Sitagliptin, a selective DDP4 inhibitor, increases caspase 3/7 activity in OC by co-treatment with paclitaxel, and maintains apoptosis induction through the ERK and Akt signaling pathway [90, 91]. These studies imply that the application of ADA and DDP4 inhibitors seems to favor tumor-cell death. However, it is interesting to note that the addition of ADA reversed the decreased metastatic capacity of OC caused by adenosine [92]. Similarly, overexpression of DDP4 leads to enhanced chemosensitivity of OC cells to paclitaxel and reduced tumor cell invasiveness due to aberrant expression of E-cadherin, MMP-2 and TIMP [93–95]. These puzzling results suggest that ADA and DDP4 still need substantial and in-depth studies and their interaction could act as potential biomarkers or therapeutic targets in OC.

Adenosine deaminases acting on RNA(ADAR, EC 3.5.4.37)catalyzes the deamidation of adenosine on dsRNA to inosine, which means A-to-I RNA editing (Fig. 3) [96]. There are three members of the ADAR family: ADAR1 and ADAR2 are primarily used for RNA editing, and ADAR3 inhibits their editing activity by binding to substrates of the first two [97–99]. ADAR-triggered aberrant RNA editing regulation to promote tumor growth is widely observed in a variety of human solid tumors [100, 101]. The frequent over-editing of Cyclin I (CCNI) by ADAR1 in OC was found to produce peptide products that activate T-cell responses and specifically kill tumor cells [21]. Marek Cybulski et al. detected that the immune responses to CCNI in the nucleus and cytoplasm of epithelial OC are related to abnormal cell cycle but not to chemosensitivity [102]. Furthermore, ADAR1 is reported to induce tumor progression by impairing Let-7d in malignant tumors [103]. Studies have found that miRNA Let-7d plays a suppressive role in a variety of human tumors and its overexpression may inhibit HMGA1 by regulating the p53 signaling pathway to enhance chemosensitivity [104–107]. These studies provide us with inspiration for gene editing to regulate tumor progression. Overall, this is a promising therapeutic concept, but the specific mechanisms still need to be explored more deeply.

### Adenylate kinase

Adenylate kinase (AK, EC 2.7.4.3) catalyzes the reversible transfer of phosphate group from ATP to AMP in purine synthesis pathway. AK-induced AMP signal changes affect adenosine pools status and energy information through metabolic sensors, which subsequently balance ATP level to regulate energy metabolism [108, 109]. AK family isozymes AK1-9 have been identified in human tissues [110], and existing studies have reported that AK1, 2, 4, 6 and 7 subtypes function in the regulation of tumor growth, metabolism, energy allocation and invasion [111–114]. So far, researches on AK in OC have been focused on AK4 and AK7.

AK4 is mainly expressed in kidney, heart and liver tissues, probably caused by the high-mitochondrial content. Significantly elevated level of AK4 is also found in some highly aggressive tumors [115–117]. Research has revealed that AK4 stabilizes the purine nucleotide pools and balances energy through the regulation of AMPK [118], and is able to interact with HIF-1 to regulate mitochondrial activity and enhance cellular hypoxia tolerance by promoting hypoxia [116, 119]. Cellular drug resistance is enhanced due to the progress in resistance to hypoxia. The interaction of hypoxia on AK4 level is different in diverse cell lines [120], however, it is clear that changes in AK4 level are able to protect cells from environmental stimuli, and blocking AK4 can resist the protective mechanism of cells against hypoxic stress [119]. AK4 can be found in oocytes, follicular epithelial cells and corpus luteum cells in normal ovary tissue [121]. AK4 is overexpressed in OC and the expression level is significantly correlated with tumor size and FIGO stage [26]. A meaningful finding is that upregulation of miRNA-3666 inhibits OC cell proliferation and migration by blocking the STAT3/AK4 axis and inducing apoptosis simultaneously [27]. Besides, AK4 favors tumor development and metastasis in an ATF3-dependent manner [122], which interestingly plays contradictory roles of either inducing apoptosis or promoting proliferation in different types of OC [123, 124].

AK7 is mainly expressed in cilia-rich sites such as respiratory tract and fallopian tube [109, 110]. Ciliary structural abnormalities caused by AK7 damage often led to primary male infertility or primary ciliary dyskinesia [125–127]. Abnormal activation or loss of primary cilia will affect the progression and prognosis of diverse tumors. Study reported that cilia-related genes are lowly expressed in glioblastoma, breast cancer, OC and colon adenocarcinoma; in contrast, they are overexpressed in clear cell renal cell carcinoma, rectal adenocarcinoma, lung adenocarcinoma and lung squamous cell carcinoma [128]. It is well known that cilia play a key role in assisting transport and picking in the female reproductive system. Furthermore, some studies support the ability of cilia as angiopoietin (Ang) sensory organ to maintain the morphological and motor homeostasis of ovarian tissue [129, 130]. Recent studies support the standpoint that the majority of OC originate from the cilia-rich fallopian tube [131] and the Ang/Tie signaling pathway associated with cilia formation may be relevant to OC development and formation [129, 132] and the expression of Ang2 is closely related to angiogenesis in OC [133]. Thus, it is reasonable to speculate that AK7 may partially influence the ability of cilia to intervene in OC progression. Zhang et al. found that AK7 level in OC is significantly reduced by analysis of the TCGA database, the degree of which is positively correlated with tumor stage [25]. And this phenomenon is mainly related to conduction pathways such as EMT, TGF-b signaling and UV response. More importantly, OC patients with lower AK7 expression have a worse prognosis. These studies not only suggest that AK7 exhibits potential as a prognostic indicator for OC but the possibility of future treatment by elevating AK7 expression level or interfering with cilia activity.

### Hypoxanthine guanine phosphoribosyltransferase

HPRT (EC 2.4.2.8) is one of the classical enzymes of the purine salvage pathway, which utilizes the transfer of ribose phosphate from PRPP to form IMP and GMP for DNA synthesis and repair [134]. As a housekeeping gene, HPRT is maintained at a low level in all somatic cells except the central nervous system [135]. HPRT deficiency leads to failure of the salvage for hypoxanthine and guanine, increasing purine degradation and UA production and triggering a series of diseases: partial deficiency causes gout-like symptoms, while complete deficiency results in Lesch-Nyhan syndrome [136]. Although other enzymes have complementary roles, these symptoms are unique to HPRT deficiency [134].

HPRT is closely associated with tumor development and is overexpressed in various malignancies. However, it has been reported that HPRT level is fluctuating in OC but stable in borderline ovarian tumor and normal ovarian tissue, making it a suitable reference gene for biochemical processes [33, 137]. HPRT performs a potential auxiliary role in DNA mismatch-repair. The reason for mismatch is that most DNA polymerases preferentially pair 7,8-dihydro-8-oxoguanine with adenine rather than cytosine during DNA oxidation [138]. The MutY DNA glycosylase homologue recognizes and repairs mismatches, whose level changes lead to opposite changes in the mutation rate of HPRT in OC cell line A2780 [139]. It implies that high-level HPRT mutation is associated with DNA mismatch activity. Nevertheless, HPRT mutation rate is reduced in OC with defects in DNA mismatch repair gene hMSH2 [140]. Also, HPRT mutation may be in connection with tumor risk and chemotherapy resistance in OC [141–145]. These contradictory results suggest that more research is needed to elucidate the relationship between HPRT mutation and DNA mismatch repair and effects on tumors. In general, current research on HPRT is focused on report gene, while tumor-related research on HPRT is relatively stagnant. In-depth investigations are still needed to explore its specific effects on tumors and therapeutic strategies.

### Inosine monophosphate dehydrogenase

The catalytic oxidation of IMP to XMP by inosine monophosphate dehydrogenase (IMPDH, EC 1.1.1.205) in cytoplasm is the NAD + -dependent rate-limiting step in de novo synthesis of GTP [146]. GTP, a classical signaling molecule that regulates various cellular activities as well as an energy supplier for protein synthesis, is elevated in a variety of tumor cells [147]. Although HPRT recovers guanines in salvage pathway, it is unable to meet the demand of malignant tumor cells. Elevated IMPDH activity enhances the ability of cancer cells to synthesize GTP, which provides raw material to supply rapid proliferation.

Abnormally high level of IMPDH is found in multiple malignancies, including OC [28, 148, 149]. Among the two isoforms of human IMPDH, IMPDH2 rather than IMPDH1 is upregulated in a variety of human tumors [29, 149–153]. Researches have reported that high expression of IMPDH2 in OC is associated with different tumor types, lower survival rates and higher stage, which implies a poorer prognosis [29]. IMPDH2 activates PI3K/Akt and Wnt/β-catenin pathways to promote progression or drive EMT processes in diverse cancers [28, 148]. The abnormally increased activity makes IMPDH, especially IMPDH2, a new target for anti-cancer drug development.

Indeed, the use of IMPDH inhibitor as an antineoplastic agent has a long history [154]. A few studies have linked IMPDH inhibitor to apoptosis: downregulation of MEK/ERK pathway leads to Bcl-2 inhibition [155]; downregulation of the Src/PI3K/Akt pathway with inhibition of mTOR to activate Bax and Bak; inhibition of mTORC1 or c-Myc signaling reduces ribosomal RNA synthesis [147, 156, 157]; drive of mitochondria-dependent mechanisms causes apoptosis [158]. Katherine Y et al. found that the addition of thiazole-4-carboxamide adenine dinucleotide, an intracellular active metabolite of tiazofurin, is effective in inhibiting IMPDH activity in OC [28]. Utilizing the IMPDH inhibitor benzamide riboside (BR) makes it possible to observe apoptosis induced by cell cycle arrest due to c-Myc and downstream Cdc25A inhibition [28, 30, 159]. Unfortunately, BR-induced apoptosis is only seen in partial OC cell lines, and the specific regulatory mechanisms between IMPDH inhibitor and apoptosis remain unknown. Furthermore, the development and application of IMPDH inhibitor has been limited by unstable effects, adverse effects at high doses, and discrepancies in IMPDH levels in different tumors [154]. For now, it is still of much value to explore IMPDH such as immunosuppression or biomarkers and this may be an opportunity to reactivate it as an antitumor agent.

### Purine nucleoside phosphorylase

**Fig. 4:**
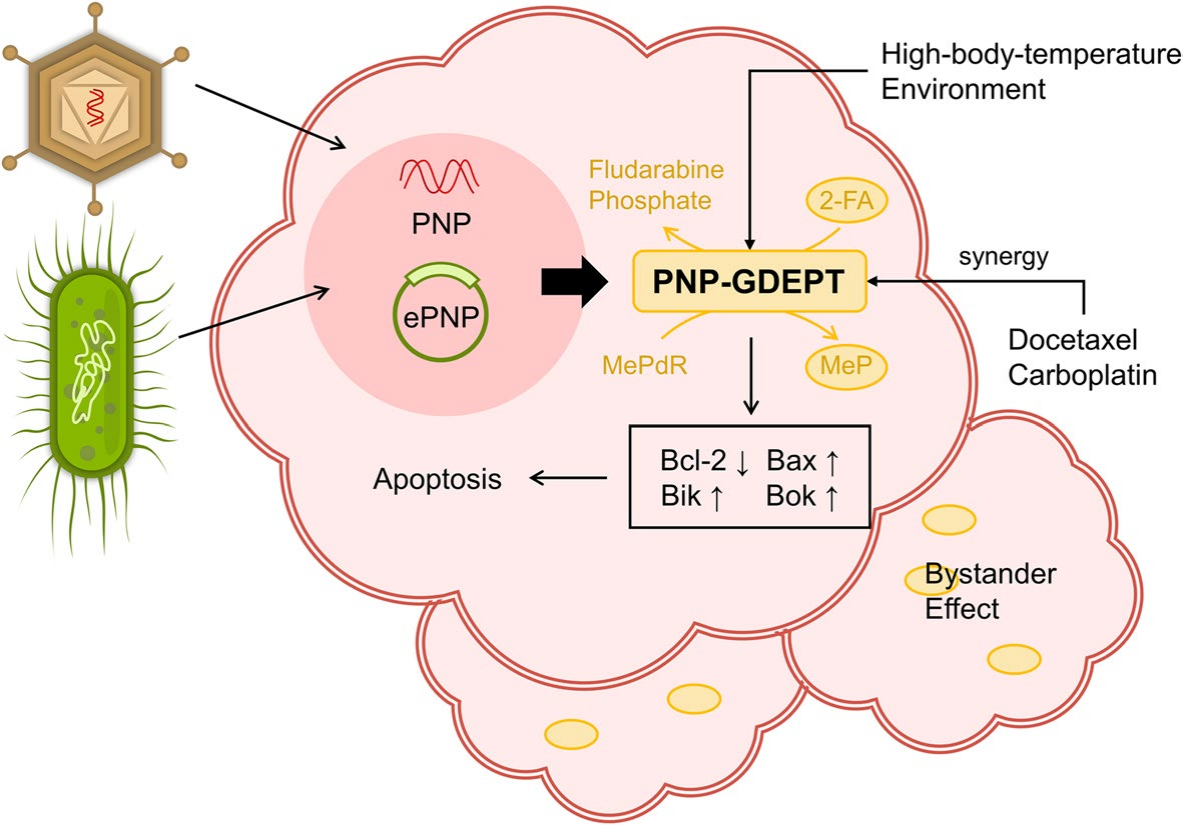
Application of PNP-GDEPT in OC. PNP cleaved MePdR and Fludarabine phosphate to the toxic products MeP and 2-FA. Implementation of GDEPT using ePNP or adenovirus-mediated PNP is able to induce apoptosis in OC cells and exert the bystander effect. PNP-GDEPT acts synergistically with docetaxel and cisplatin and high-body-temperature environment enhance the expression efficiency of ePNP. GDEPT: gene directed enzyme prodrug therapy; ePNP: E. coli PNP; MePdR: 6-methylpurine-2’-deoxyriboside; MeP: 6-methylpurine; 2-FA: 2-Fluoroadenine

Purine nucleoside phosphorylase (PNP, EC 2.4.2.1), which catalyzes the reversible phosphorylation of adenosine, guanosine and inosine, is an important enzyme in salvage and degradation pathway [32]. Among them, homologous E. coli PNP (ePNP) rather than human PNP is able to enrich purine pools with adenosine as a substrate [160]. Gene directed enzyme prodrug therapy (GDEPT), also known as suicide gene therapy, relies on transgenic methods to encode enzymes that convert non-toxic drug precursors into active toxic metabolites to target tumor cells injury [161]. The anti-tumor effect of selective expression of the suicide gene PNP has been observed in a variety of malignancy researches (Fig. 4) [162, 163]. PNP-GDEPT exerts anti-tumor activity in a unique way compared to others. It cleaves 6-methylpurine-2'-deoxyriboside and fludarabine phosphate to toxic purine analogs 6-methylpurine and 2-Fluoroadenine, which inhibits nucleic acid and protein synthesis [164]. These toxins are then released into extracellular matrix and remain active [32]. That is, toxic metabolites can spread to surroundings to achieve significant bystander killing effect even if only few tumor cells express ePNP, which suggests a potential chemotherapeutic advantage [165, 166].

A few attempts of this approach have been tested in OC cells. V K Gadi et al. have successfully modeled ePNP bystander killing which exerts significant antitumor effects of OC in vitro and in vivo [166]. Other researchers reported that adenovirus-mediated PNP-GDEPT caused upregulation of Bax, Bik and Bok and downregulation of Bcl-2 [167]. Meanwhile, there was synergy between PNP-GDEPT and docetaxel or carboplatin on platinum-resistant OC cells, implying the potential for reversing drug resistance. Unfortunately, this approach exhibited a low success rate. In this regard, researchers combined PNP-GDEPT with high-body-temperature environment created by human telomerase reverse transcriptase and heat shock elements to improve PNP expression efficiency [168]. Thus, while novel evidence is currently limited, these investigations may provide a new strategy for GDEPT in OC.

### Other purine-metabolizing enzyme

**Fig. 5:**
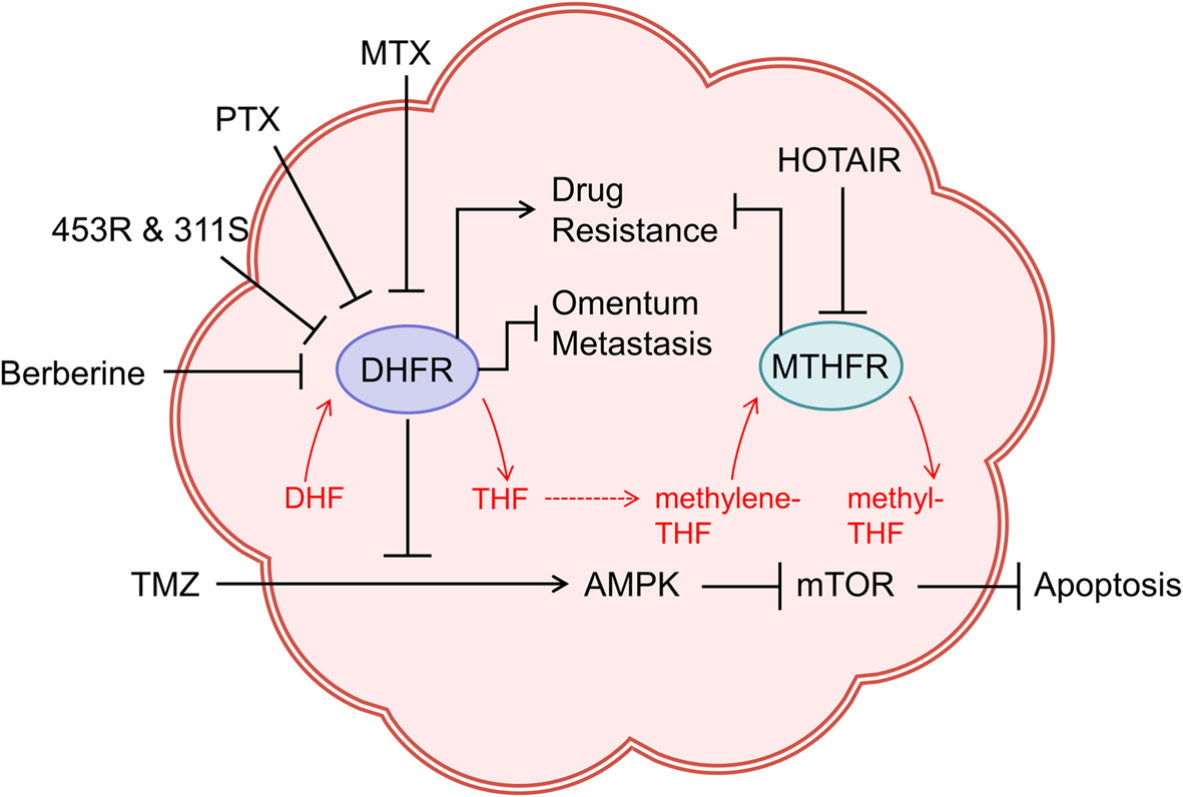
Role of DHFR and MTHFR in OC. DHFR and MTHFR are involved in the formation of important one-carbon units for purine metabolism. DHFR promotes drug resistance and inhibits omentum metastasis, while resisting apoptosis caused by TMZ through AMPK pathway activation and mTOR pathway inhibition. Berberine, PTX, MTX, and some quinoxalines (453R&311S) have been found to act as DHFR inhibitors. MTHFR inhibits FBP expression and enhances drug sensitivity, which is inhibited by HOTAIR. TMZ: temozolomide; PTX: pemetrexed; MTX: methotrexate; 453R: 3-methyl-7-trifluoromethyl-2(R)-[3,4,5-trimethoxyanilino]-quinoxaline; 311S: 3-piperazinilmethyl-2[4(oxymethyl)-phenoxy]-quinoxaline; FBP: folate binding protein

Dihydrofolate reductase (DHFR, EC 1.5.1.3) and 5,10-methylenetetrahydrofolate reductase (MTHFR, EC 1.5.1.20) are not conventional purine metabolizing enzymes as they control the metabolism of purines and pyrimidines indirectly by regulating folate synthesis (Fig. 5). Folate acts as a raw material and coenzyme as well as a methyl donor in the biosynthesis of nucleotides [169]. DHFR reduces dihydrofolate to tetrahydrofolate (THF) which subsequently acquires one-carbon units from amino acids to install methyl groups [170]. MTHFR continues to convert methylene-THF to methyl-THF for further participation in nucleic acid biosynthesis. In fact, methyl-THF, methylene-THF and formyl-THF are interconverted to provide one-carbon units for methionine cycle pathway, thymidylate synthesis pathway and purine synthesis pathway in turn [171]. Studies have reported that folate-mediated one-carbon metabolism plays an important supporting role for rapidly proliferating cells, especially tumor cells [169] and DNA methylation is expected to be used as an early diagnostic marker for various malignancies [172–174]. Overall, folate metabolism, the upstream of purine metabolism, serves an important role in tumor development.

Multiple studies have shown that the overexpression of DHFR is associated with platinum resistance in OC [36–38]. Interestingly, Jia Chen et al. reported that DHFR expression is increased in benign ovarian tumor but decreased in malignant OC with a significant correlation with omentum metastasis [39]. In this case, however, DHFR expression is still significantly higher in platinum-resistant patients than platinum-sensitive patients, highlighting the potential diagnostic value of DHFR in chemoresistance. Actually, the commonly used chemotherapy drug methotrexate (MTX) targets DHFR to block THF production to inhibit rapidly proliferating tumor cells [175]; however, given its toxicity and resistance, there has been a search for new DHFR inhibitors. Pemetrexed, a DHFR and thymidylate synthase inhibitor, activates AMPK and inhibits mTOR signaling pathway by synergizing with temozolomide (TMZ) to obstruct tumor growth [176]. Berberine disturbs folate-metabolizing enzymes including DHFR to reduce the viability of OC cells, especially platinum-resistant cells. In addition, there is evidence that some compounds with quinoxaline structures are not cross-resistant with cisplatin and show remarkably inhibition on OC growth, demonstrating the potential as DHFR inhibitors [37, 177].

As a rate-limiting enzyme for folate metabolism, MTHFR with low viability slows down the synthesis and repair of nucleotides and disrupts the methylation of homocysteine [178, 179]. Defective MTHFR in OC combined with overexpression of high-affinity folate-binding proteins lead to increased folate uptake, implying that low-activity MTHFR is a potential risk factor for OC [40]. Current studies have found that MTHFR single nucleotide polymorphisms (SNPs), particularly C677T polymorphism which affects enzyme activity, are likely to increase tumor risk [41, 180]. Many studies on the association of C677T with susceptibility and risk of OC in Asians rather than whites have been performed [181–184]. However, no effect of C677T polymorphism in Asians was found in a 2012 meta-analysis, possibly because of the limitation of sample size [42]. A possible association between high folic acid intake and low patient survival was reported in research by S C Dixon et al. [43]. However, an interesting finding is that a reduced risk of OC in Chinese with high folate intake was more pronounced in MTHFR 677CC mutation, but no such protection was observed in Australians [185, 186]. This discrepancy could be attributed to the limitations of the sample and ethnicity, emphasizing the need for more research. Recently investigators have examined the effect of MTHFR on the efficacy of 5-fluorouracil (5-FU) chemotherapy in OC patients. Silencing HOX transcript antisense RNA elevates the sensitivity to 5-FU due to decreased MTHFR methylation [187]. Besides, MTHFR SNP has been found correlated with prognosis or hematologic toxicity in 5-FU-treated patients in other human tumors [188–190]. Yet research on this link in OC has remained an under-explored domain and more in-depth explorations are needed.

## Purinergic signaling in OC

### Key agonist, eATP and eADO

The extracellular purine, mainly eATP and eADO, has active traffic with intracellular signals. In normal physiological environments, high eATP and low eADO levels are maintained through exocytosis or vesicles [191]. Nevertheless, the overall level is subject to strict limitations [192]. The eATP concentration increases dramatically with intracellular ATP in disordered TME (even up to two times the intracellular concentration), which may be due to active release from tumor cells or immune cells, or to abnormal metabolites. As previously described, CD39 and CD73 contribute to the progressive dephosphorylation of eATP to eADO. Continuous stimulation of ATP causes active phospholipase D to promote ATP degradation [193]. Meanwhile, hypoxia effectively stimulates adenosine release [194–196], possibly due to HIF-1α-induced adenosine kinase inhibition that impedes the conversion of ADO to AMP and thus promotes its efflux [197], or possibly as a consequence of retaliatory metabolism [196]. The cascade response results in abnormal levels of both intra- and extracellular ATP and ADO to regulate tumor development via purinergic signaling as autocrine or paracrine messengers [13].

**Fig. 6:**
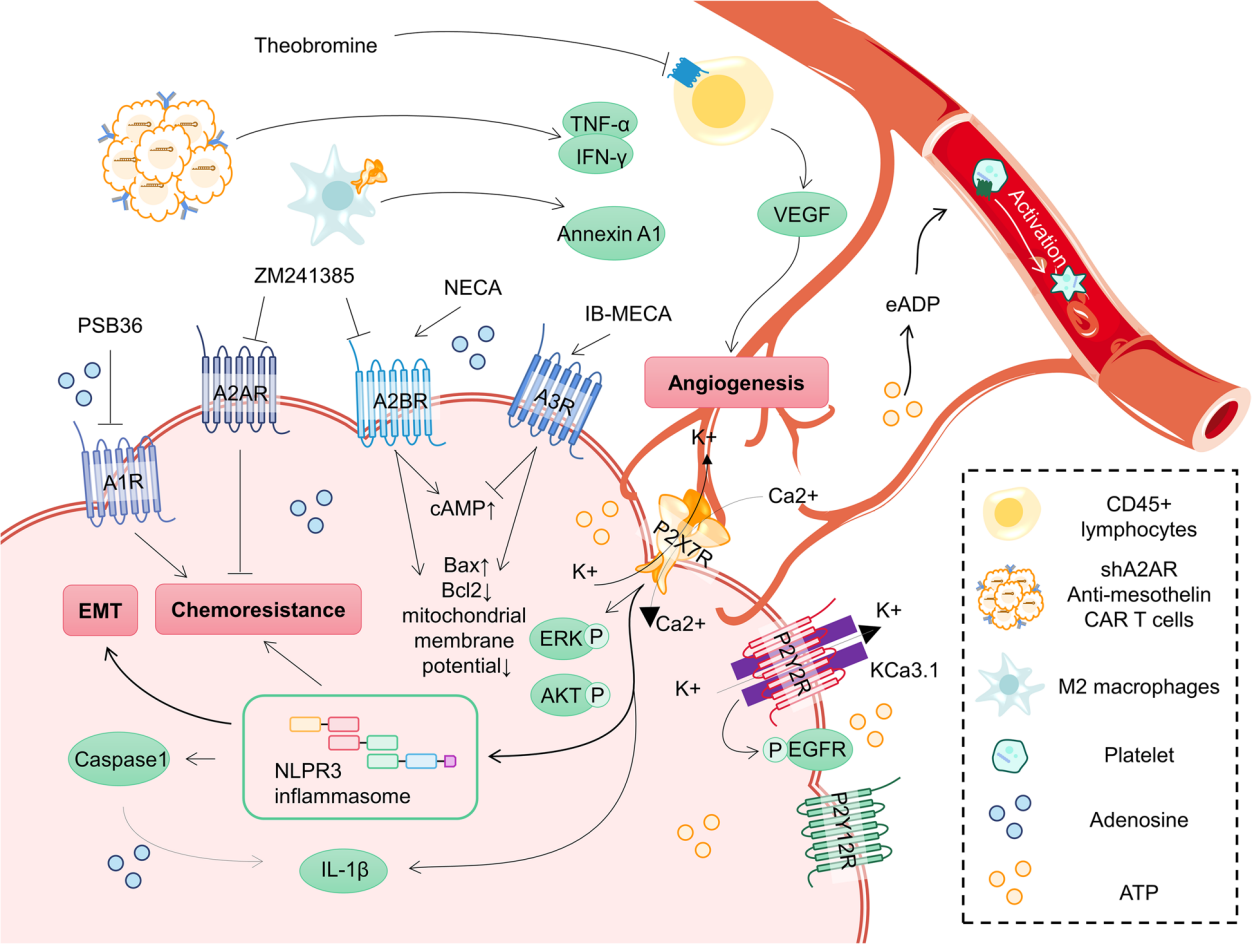
Purinergic signaling pathway in OC. Extra- and intracellular adenosine and ATP are key agonists. Purinergic receptors are expressed in a variety of cells in OC TME. Activation or antagonism of these receptors, as well as interaction with other signaling will ultimately affect the progression and malignant features of OC

Generally, eATP and eADO are considered as important signals to regulate immunity with pro-inflammatory and anti-inflammatory effects, respectively [198, 199]. The sharp increase in eADO hinders immune cell activation to exert pro-cancer effects and reduces survival of OC patients [200–203]. This effect is closely related to purinergic receptors, which will be described later (Fig. 6). For other malignant features of ovarian cancer, eATP and eADO also play see-saw roles. The eATP activates corresponding purinergic receptors and its co-expressed KCa3.1 channel, triggering subsequent complex electrical membrane responses that contribute to cancer migration [204]. Conversely, eADO supplementation or its analogs have been reported to inhibit the ability of OC to invasion or angiogenesis, partially dependent on the promotion of RhoGDI2 subsequently related gene expression [205, 206]. The effect of adenosine analog supplementation on OC chemoresistance likewise depends on the type of receptor activated [207]. Notably, low doses of adenosine were associated with G0/G1 phase arrest, whereas high concentrations of adenosine induced both early and late apoptosis in a dose-dependent manner by activating the Bcl-2/Bax and caspase-3 pathway in OC cells [208]. These complex effects shift our thinking to future studies of eATP/eADP ratios rather than single substance levels.

### Purinergic receptors and their therapeutic potential

Previous studies have shown that purinergic receptors are expressed in almost all immune cells, and an increasing number of studies have identified the presence of purinergic receptors in tumor cells [209]. The identified purinergic receptors include P1 receptors that mediate signals triggered by adenosine and P2 receptors that respond to adenine and uridine nucleotides. In general, P1 receptors and P2 receptors mediate pro-inflammatory and anti-inflammatory responses, respectively, which should actually be more dependent on specific receptor types and signaling transduction ratios [210].

#### P1 receptors

P1 receptors, also known as adenosine receptors, are divided into four subtypes, A1R, A2AR, A2BR and A3R, of which A2R is a key factor in protecting tissues from excessive immune responses and commonly expressed in a variety of human tumours [211–214]. A2AR is a high-affinity receptor involved in the regulation of T-cell function to promote immune evasion, and inhibits the secretion of defensive substances by neutrophils to attenuate the inflammatory response [54, 215]. A2BR appears to have low affinity and is more expressed in macrophages and dendritic cells. Another study found that activation of A2BR was able to interfere with TME via MDSCs to promote cancer growth [216]. Besides, the high-affinity receptors A1R and A3R are considered as immune-promoting adenosine receptors, possibly because of promoting IL-10 expression or inhibiting cAMP production [217, 218]. Therefore, adenosine receptors deserve to be another target of intervention in tumor immunotherapy and hold considerable promise.

A2BR and A3R are the predominantly expressed adenosine receptors in OC [219]. It has been reported that activation of A2BR significantly increases cAMP levels in OC, while activation of A3R exerts the opposite effect, and the other two receptors do not affect cAMP levels, implying that A2BR and A3R are major functional adenosine receptors in OC. In addition, A2BR activation significantly inhibits the migration ability of OC and maintains the epithelioid phenotype [220]. Kaplan–Meier survival analyses showed that OC patients with low A2BR expression levels, especially with early-stage OC, had shorter OS. Interestingly, however, Hajiahmadi et al. found that either the A2BR agonist NECA or the A3R agonist IB-MECA dose-dependently inhibited OC cell viability in a manner that related to the loss of mitochondrial membrane potential to activate apoptosis [221, 222]. Although these findings reflect the potential OC-inhibitory effect of activating A2R and A3R, changes in cAMP, which link essential malignant features such as invasion, metastasis, apoptosis resistance and chemoresistance, are not mentioned [223–225]. The non-dominantly expressed adenosine receptor, A2AR, plays a role in assisting chimeric antigen receptor (CAR) T cells from hindrance by the immunosuppressive microenvironment [226]. Liu G et al. found that anti-mesothelin CAR T cells released more TNF-α and IFN-γ after suppressing A2AR, and extremely enhanced anti-OC efficacy, which provided a new perspective for OC treatment [227]. It should be emphasized that the complex regulatory mechanisms and effects are still not completely clear because different cell status, receptor types, and environmental conditions may cause influences, and further studies to identify the pro- or anti-cancer roles played by adenosine receptors and the specific mechanisms are urgent and necessary.

In fact, targeting adenosine receptors has shown anticancer therapeutic potential in OC. Theobromine, a non-selective adenosine receptor antagonist, inhibits OC angiogenic activity by reducing vascular endothelial growth factor production [228]. Further studies revealed that the inhibition of angiogenesis was associated with A2BR interaction of CD45 lymphocytes [229, 230]. However, blind application of the adenosine analogue ZM241385, an A2R antagonist, may increase the resistance to cisplatin [207], and the A1R antagonist PSB36 brought a sensitizing effect. Overall, activation of adenosine receptors can be anti- and pro-cancer, and the opposing efficacy of their dual action may bring valuable insights for future clinical therapeutic interventions with further studies.

#### P2 receptors

P2 receptors consist of two subfamilies, P2XRs and P2YRs. P2XRs are ATP-gated non-selective cationic conductance channels with seven isoforms (P2X1-7R) that are expressed in a variety of tumor cells, immune cells, and stromal cells [209]. The eATP is a recognized agonist, and some non-nucleotide compounds have been reported to modulate it as well [231–233]. P2XRs, especially P2X7R, are the ones that have been focused on for their multiple roles in mediating tumor growth, metastasis, invasion, drug resistance, and death, in addition to pro- and anti-inflammatory effects [234–238]. Overexpression of P2X7R in OC contributes to cell proliferation and viability [239, 240]. Vázquez-Cuevas FG et al. found that activation of P2X7R caused an increase in intracellular Ca(2+) concentration and phosphorylation of ERK and AKT, but did not cause apoptosis [241]. Notably, eATP, or actually mainly the activation of P2X7R, promotes NLPR3 inflammasome activation and assembly, followed by activation of caspase 1, which is dependent on ATP induction and subsequently K(+) efflux [75]. Meanwhile, P2X7R drives IL-1β maturation in response to activation of the NLRP3 inflammasome [242]. It was shown that excessive NLRP3 in OC promotes EMT [243, 244] and mediates gemcitabine resistance [245] through the Wnt/β-catenin signaling pathway, and is associated with poorer OS [246]. Once NLRP3 is absent, the expression of P2X7R will be promoted, which contributes to cancer growth as a negative feedback loop [247]. Co-localization of P2X7R/NLRP3 in the adipocyte plasma membrane of omental tissue of OC patients implies its possible contribution to tumor metastasis [248]. However, it is noteworthy that antagonizing P2X7R inhibits pyroptosis via NLRP3/caspase1 and promotes apoptosis mediated by Bcl-2/caspase9/caspase3 pathway at the same time [249]. In addition, P2X7R stimulation in alternative activated M2 macrophages has been reported to release abundant anti-inflammatory proteins, suggesting a contribution to the inflammation resolution [238]. The P2X7R/NLRP3 complex lacks detailed studies in OC, and the pro- and anti-inflammatory and even pro- and anti-cancer responses it mediates are incompletely understood as well. The balance between mutually inhibited pyroptosis and apoptosis may bring a fresh perspective to precisely fight OC.

P2YRs are G protein-coupled and contain a total of eight isoforms (P2Y1R, P2Y2R, P2Y4R, P2Y6R, P2Y11R, P2Y12R, P2Y13R and P2Y14R) in mammals. Compared to P2XRs, P2YRs are more sensitive to slight changes in local nucleotide or agonist concentrations [250]. These members are activated by different nucleotides, and only P2Y2R and P2Y11R are responsive to ATP [251]. The prominent contribution of P2YRs in tumor growth and metastasis cannot be ignored, which is associated with the regulation of intracellular Ca(2+) concentration and subsequently cAMP changes. In this regard, P2Y1R, P2Y2R, P2Y4R, P2Y6R bind to Gq-dominated protein subunits to activate PLCβ/IP3/DAG signaling pathway to increase intracellular Ca(2+) concentration, which may be affected by the acidic intracellular environment [251, 252]. P2Y12-14R couple to Gi/o to inhibit adenylate cyclase activity to reduce intracellular cAMP levels. As previously described, low levels of ATP stimulate P2Y2R, and activate co-localized KCa3.1 channels to promote migration of OC cells [204]. Martínez-Ramírez AS et al. suggested that the contribution of P2Y2R to OC cell migration may derive from an interaction with EGFR [253]. In addition, there is an increasing number of studies reporting the special status of platelets in OC. OC cells induce platelets activation, and in turn, platelets stimulate OC growth [254, 255]. Cho MS et al. revealed that activation of P2Y12R on platelets by ticagrelor contributes to OC growth, for which eADP secreted by OC cells is an important activator [256]. P2Y2R and P2Y12R may play as pivots in OC progression, however, more specific mechanisms need to be further investigated.

Altogether, the understanding of purinergic receptors in OC is currently incompletely clear. More in-depth studies need to be conducted to map a detailed purinergic signaling network in OC to provide novel insights for precise treatment.

## Purine antimetabolites

Purine antimetabolites are one of the classical approaches to antitumor, which are chemical analogues of purine metabolism substrates and block purine metabolism by two mechanisms: mimic physiological substrates and compete for the same metabolic enzyme binding site to interfere with biochemical reaction rates resulting in reduced production of normal metabolites [257]; bind to the active site and generate inactive or even toxic metabolites which cause DNA damage and induce apoptosis [258]. Purine antimetabolites are mainly classified into thiopurines and purine deoxynucleoside analogues according to the structure and mechanism [257]. Current researches on OC focuses on 6-thioguanine (6-TG), 6-mercaptopurine (6-MP), MTX and fludarabine (Table 2). Besides, here we classify clopidogrel, which targets purinergic receptors, as an atypical purine antimetabolite.

**Table 2:** The basic information and effects of 6-thioguanine, 6-mercaptopurine, methotrexate, fludarabine and clopidogrel in OC

	CAS number	Molecular Formula	Target	FDA-Approved Date	Effects in OC	Ref
6-Thioguanine	154–42-7	C_5_H_5_N_5_S	PRPP amidotransferase	1966	Not exactly	[259]
6-Mercaptopurine	50–44-2	C_5_H_4_N_4_S	PRPP amidotransferase	1953	Antitumor	[260]
Methotrexate	59–05-2	C_20_H_22_N_8_O_5_	DHFR	1971	Antitumor	[261, 262]
Fludarabine	21,679–14-1	C_10_H_12_FN_5_O_4_	Nucleotide reductase; DNA polymerase; DNA ligase	1991	Antitumor	[263–266]
Clopidogrel	113,665–84-2	C_16_H_16_ClNO_2_S	P2Y12R (in platelets)	1997	Not exactly	[267–269]

6-TG is a guanosine analogue that is converted by HPRT to toxic 6-thioguanine nucleotides, which exert pharmacological effects by binding to DNA [11]. It has been suggested that 6-TG may be an effective therapeutic agent for BRCA-deficient tumors, especially for platinum-resistant and PARP inhibitor-resistant tumors [259]. This is due to the fact that homologous recombination is reactivated in resistant cells without complete recovery from the damage caused by 6-TG. Unfortunately, long-term treatment is not recommended because of its hepatotoxicity [270]. 6-MP is a structural analogue of hypoxanthine with a similar mechanism as 6-TG and a lower toxicity [271]. After treatment with 6-MP and MTX for two months, 30% of OC patients with BRCA mutations showed stable disease status afterwards, and 14% showed longer-term clinical benefit [260]. Nevertheless, neutropenia and anemia are the most common adverse effects. A phase II clinical trial continues to evaluate the safety of 6-MP, which may provide an attack on BRCA-deficient OC [270]. The mechanism of MTX resistance to purine metabolism was described above. MTX was recently reported to induce apoptosis by increasing ROS, inducing DNA damage and modulating mitochondrial membrane potential [261]. Oral low-dose MTX and cyclophosphamide may serve as maintenance therapy after chemotherapy for patients with advanced OC [272]. Furthermore, Courtney A Penn et al. found that therapeutic combination of MTX and nanoparticle targeting TAM for OC exhibited superior activity over cisplatin alone [262]. At the same time, MTX ovarian toxicity cannot be ignored [273]. As for fludarabine, a fluorinated nucleotide analogue of vidarabine, is relatively resistant to ADA inactivation [274]. It is first converted to 9-β-D-arabino-furanosyl-2-fluoradenine (F-ara-A) that can be taken up by cells, and then to F-ara-A triphosphate which inhibits nucleotide reductase, DNA polymerase and DNA ligase, and ultimately causes impaired DNA synthesis and apoptosis [12]. Fludarabine was found to reduce OC migration and adhesion by inhibiting the FAK/STAT1 pathway [263] and was able to inhibit VEGF via Hif-1α and PI3K/AKT signaling pathways to arrest the progression of OC [264]. Moreover, it exerts synergistic effects with cisplatin, seemingly supporting its potential as a modulator of chemotherapeutic agents [265]. Two clinical trials utilize fludarabine as an immunosuppressant to deplete lymphocytes for allogeneic NK cell therapy in OC patients [275, 276]. There were also some important differences in other purine antimetabolites that contribute to the development of OC [277]. These conflicting roles indicate the need for more research and clinical trials on purine antimetabolites in OC. Additionally, interventions on purinergic signaling are the result of atypical purine antimetabolites, which lack sufficient evidence for therapeutic trials in OC. High-dose clopidogrel has been shown to cause high-level P2Y12 blockade [267]. A concern is that clopidogrel may increase the risk of toxicity when used with paclitaxel, as was found in a 60-year-old patient with OC [268]. A higher risk is also seen in the report by Park SH et al. [269]. It remains to be considered whether interventions on purinergic signaling pathways can provide benefits to OC patients.

## Conclusions

Homeostasis of purine pools in vitro and in vivo is essential for the maintenance of healthy state and normal function of cells. The depletion of purine in normal cells can be saved by an increase in purine synthesis [278]. In some cases, excessive purine consumption is beyond the capacity of synthesis and is unable to be compensated. For example, excessive DNA synthesis in S1 phase leads to a dramatic increase in purine consumption, or a plethora of purine neurotransmitters are released during the active phase of certain neurons [279]. Imbalance of purine pools contributes to dysregulation of genomic stability and ultimately to metabolic disease or tumor development [280, 281]. Herein, we summarize the role of major purine-metabolizing enzymes, describe purinergic signaling pathways, outline the roles and mechanisms of partial purine antimetabolites and point out potential therapeutic strategies for targeting purine metabolism in OC.

In fact, purine-metabolizing enzymes also participate in other biological processes. For instance, CD39 and ADA are involved in immune regulation [18, 58, 59, 83]; IMPDH carries out the function of a transcription factor [282]; SAM Domain And HD Domain-Containing Protein-1 assists in regulating the cell cycle [283] and phosphoribosylaminoimidazole succinocarboxamide synthetase is correlated with DNA damage repair [284, 285]. It can help to identify new interventions in tumor process if we focus on purine metabolic processes and understand the mechanisms of action of the relevant enzymes as well as the interaction with other biological processes. Additionally, uncontrolled tumor proliferation due to excessive purine synthesis plays a role in chemoresistance, which may be sensitized by inhibitors of purine metabolism [286]. Purine antimetabolites and other anti-purine-metabolizing agents may provide an additional line of attack that would be a promising strategy to overcome tumor resistance in combination with conventional chemotherapy.

However, it cannot be denied that purine metabolism in OC has not been comprehensively studied and is still at a relatively superficial stage. So far, most studies have only briefly described purine-metabolizing enzyme changes and subsequent cell apoptosis, without exploring the relationship and mechanisms between the two in detail. Several studies even showed contradictory treatment results, probably caused by the lack of samples. Evidence above casts doubt on the credibility of the link between the two. Besides, it is difficult to determine whether adenosine disorders are caused by enzyme effect or an action of Adenosine itself, or are related to adenosine receptors. Likewise, it is a critical option to activate or antagonize purinergic receptors in OC. The interaction of purinergic signaling with hypoxia or NLRP3 inflammasomes is also a valuable reflection on how to intervene to obtain maximum benefit. Another limitation is that basic researches on OC treatment options targeting purine metabolizing enzymes or purinergic signaling pathways are currently stagnant and lack the support of clinical research results. Therefore, it is necessary to focus on the association of purine metabolism with other human tumors, which may open up new possibilities for OC research.

Recent studies have reported that multiple kinases interfere with tumor progression by affecting purine de novo pathway via regulation of important transcription factors or intervention with rate-limiting enzymes: DYRK3 regulates ATF4 transcriptional activity and inhibits PPAT to suppress hepatocellular carcinoma proliferation and metastasis [287]; UHMK1 modulates the NCOA3/ATF4 axis and may activates ATIC to promote gastric cancer development [288]; CLK3 stabilizes the USP13/Fbxl14/c-Myc axis to enhance cholangiocarcinoma aggressiveness [289]. These outcomes broaden the horizon, including targets beyond the enzymes that are directly involved in purine metabolism.

In any case, more researches are needed to understand the mechanisms of aberrant purine metabolism in OC. In-depth knowledge of purine metabolic processes may help to better understand cancer-related metabolic reprogramming and develop new inhibitors accordingly, which presents exciting opportunities for OC therapy. We will investigate this in more depth in the future and believe that this may become a promising novel strategy for OC therapy. 

## Data Availability

Not applicable.

## Abbreviations

ADA: Adenosine deaminase
ADAR: Adenosine deaminases acting on RNA
ADSS: Adenylosuccinate synthase
AK: Adenylate kinase
Ang: Angiopoietin
APRT: Adenine phosphoribosyltransferase
BR: Benzamide riboside
CAR: Chimeric antigen receptor
CCNI: Cyclin I
CD39: Ectonucleoside triphosphate diphosphohydrolase
CD73: Ectosolic-5′-nucleotidase
DDP4: Dipeptidyl peptidase 4
DHFR: Dihydrofolate reductase
eADO: Extracellular adenosine
eAMP: Extracellular AMP
eATP: Extracellular ATP
EMT: Epithelial–mesenchymal transition
ePNP: E. coli PNP
F-ara-A: 9-β-D-arabino-furanosyl-2-fluoradenine
GDEPT: Gene directed enzyme prodrug therapy
HPRT: Hypoxanthine guanine phosphoribosyltransferase
IMP: Inosine monophosphate
IMPDH: Inosine monophosphate dehydrogenase
MDSC: Myeloid-deriver suppressor cell
MTHFR: 5,10-Methylenetetrahydrofolate reductase
MTX: Methotrexate
OC: Ovarian cancer
PD-1: Programmed death-1
PNP: Purine nucleoside phosphorylase
PRPP: 5-Phosphoribosyl-1-pyrophosphate
SNP: Single nucleotide polymorphisms
TAM: Tumor-associated macrophage
THF: Tetrahydrofolate
TME: Tumor microenvironment
TMZ: Temozolomide
UA: Uric acid
XMP: Xanthosine monophosphate
XO: Xanthine oxidase
5-FU: 5-Fluorouracil
6-MP: 6-Mercaptopurine
6-TG: 6-Thioguanine
